# Supplementary material for: Identification of photoperiod-induced specific miRNAs in the adrenal glands of Sunite sheep (Ovis aries)
Source: Front Vet Sci. 2022 Jul 22;9:888207. doi: 10.3389/fvets.2022.888207 (PMC9354845; doi:10.3389/fvets.2022.888207)
Supplement: Supplementary file 1 [file Data_Sheet_1.ZIP › Supplementary file/miRDeep2 file.zip!/miRDeep2 file/Novel_Structure/SPLP42A1/Novel_70.pdf]

Provisional ID	:	NC_019476.2_25472
Score total	:	2.4
Score for star read(s)	:	-1.3
Score for read counts	:	0
Score for mfe	:	2.1
Score for randfold	:	1.6
Score for cons. seed	:	
Total read count	:	142
Mature read count	:	132
Loop read count	:	0
Star read count	:	10

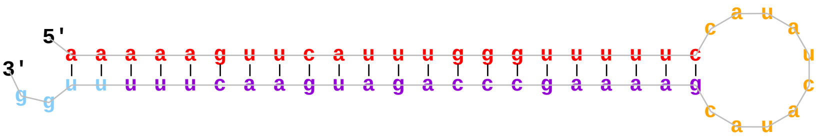

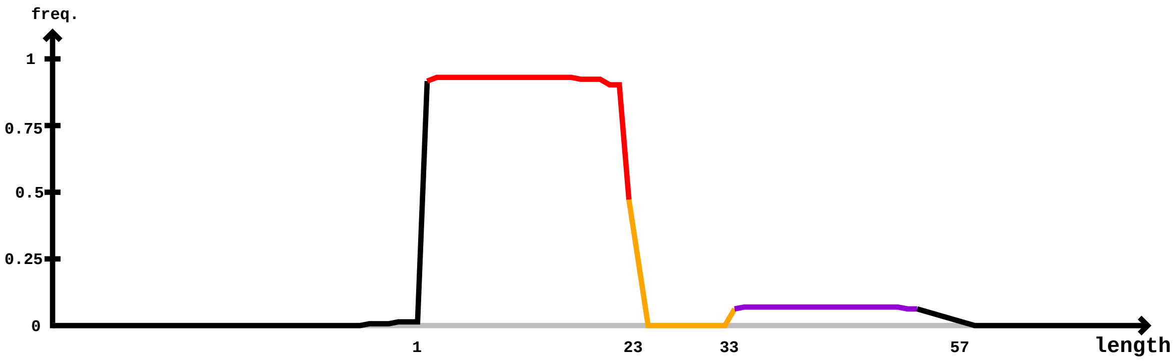

Mature   Star

```
5'- gagcaggagaagcauacaaauagguauuggguuggucaaaaaguucauuuggguuuuuccauaucauacgaaaagcccagaugaacuuuuuggucaauccaguauuaaaacc   -3'   obs
    gagcaggagaagcauacaaauagguauuggguuggucaaaaaguucauuuggguuuuuccauaucauacgaaagcccagaugaacuuuuuggucaauccaguauuaaaacc         exp
    ..((.......)).........(((((((((((((((((((((((((((((((((((((..........)))))))))))))))))))))))))))))))))))))......   reads   mm   sample
    ...............................uuggGcaaaaaguucauuuggg...........................................................   1       1    seq
    ..................................gucaaaaaAuucauuuggguuu........................................................   1       1    seq
    .....................................aaaaaguucauuuggguuu........................................................   1       0    seq
    .....................................aaaaaguucGuuuggguuuuu......................................................   62      1    seq
    .....................................aaaaaguucGuuuggguuuuuc.....................................................   66      1    seq
    .....................................aaaaaguucauuuggguuuuucU....................................................   1       1    seq
    ......................................Uaaaguucauuuggguuu........................................................   1       1    seq
    ......................................aaaaguucauuuggguuuuucU....................................................   1       1    seq
    .....................................................................gaaaaAcccagaugaacu.........................   1       1    seq
    .....................................................................gaaaaAcccagaugaacuuu.......................   8       1    seq
    ......................................................................aaaagcccagaugaacuuuuug....................   1       0    seq
```
